# Host-Bacteria Crosstalk at the Dentogingival Junction

**DOI:** 10.1155/2012/821383

**Published:** 2012-07-26

**Authors:** M. T. Pöllänen, M. A. Laine, R. Ihalin, V.-J. Uitto

**Affiliations:** ^1^Department of Periodontology, Institute of Dentistry, University of Turku, Lemminkäisenkatu 2, 20520 Turku, Finland; ^2^Department of Cariology, Institute of Dentistry, University of Turku, Lemminkäisenkatu 2, 20520 Turku, Finland; ^3^Department of Biochemistry and Food Chemistry, University of Turku, 20520 Turku, Finland; ^4^Department of Periodontology, Institute of Dentistry, University of Helsinki, 00014 Helsinki, Finland; ^5^Department of Oral and Maxillofacial Surgery, Helsinki University Central Hospital, 00290 Helsinki, Finland

## Abstract

The dentogingival junction is of crucial importance in periodontal host defense both structurally and functionally. Oral bacteria exert a constant challenge to the host cells and tissues at the dentogingival junction. The host response is set up to eliminate the pathogens by the innate and adaptive defense mechanisms. In health, the commensal bacteria and the host defense mechanisms are in a dynamic steady state. During periodontal disease progression, the dental bacterial plaque, junctional epithelium (JE), inflammatory cells, connective tissue, and bone all go through a series of changes. The tissue homeostasis is turned into tissue destruction and progression of periodontitis. The classical study of Slots showed that in the bacterial plaque, the most remarkable change is the shift from gram-positive aerobic and facultatively anaerobic flora to a predominantly gram-negative and anaerobic flora. This has been later confirmed by several other studies. Furthermore, not only the shift of the bacterial flora to a more pathogenic one, but also bacterial growth as a biofilm on the tooth surface, allows the bacteria to communicate with each other and exert their virulence aimed at favoring their growth. This paper focuses on host-bacteria crosstalk at the dentogingival junction and the models studying it *in vitro*.

## 1. Microorganisms in Subgingival Biofilm

The presence of the initial colonizers on the tooth surface is essential for the emergence of the gram-negative periodontopathogens *Porphyromonas gingivalis, Tannerella forsythia (Bacteroides forsythus)*, *Aggregatibacter actinomycetemcomitans,* and *Fusobacterium, Prevotella, Campylobacter, *and *Treponema *species [1–3]. Recently also gram-positive genera such as *Peptostreptococcus* and *Filifactor* have been suggested to play roles in periodontitis [4]. The genera *Megasphaera* and *Desulfobulbus* have also been found to be elevated in periodontitis, even to a larger extent than species traditionally implicated as periodontopathogens [4]. Recent studies have also identified *Staphylococcus aureus* in pockets of the majority (60,5%) of nonsmoking patients with aggressive periodontitis [5]. In addition to known periodontal pathogens, other bacterial species, such as *Pseudoramibacter, Bacteroidetes, Sphorocytophaga, Shuttleworthia, Dialister, Mogibacterium, Mycoplasma, Synergistes, *and* Acidaminococcaceae*, seem to exist at high levels in patients with refractory nature of periodontitis [6].

Microorganisms other than bacteria have also been found in periodontitis patients. Candida species have been detected in 15–21% of periodontitis patients as well as in healthy subjects [3, 7–9]. The coexistence of, for example, Epstein-Barr and human cytomegalovirus together with periodontopathogens has also been implicated to play a role in periodontal pathogenesis [10].

Although novel detection methods have allowed the recognition of new species that could be involved in the pathogenesis of periodontitis, recent studies suggest that the whole composition, rather than single species, influence the pathogenicity of the biofilm [11, 12]. In addition to the protective structures of multispecies biofilm which inhibit the actions of host defense cells, the molecular interactions between different species [13, 14] in the oral biofilm could influence the virulence of the bacterial community. Especially quorum sensing signal, autoinducer-2, is used in inter-species signaling between various periodontal pathogens, and it may enhance the formation of the biofilm as well as change the virulence gene expression [15]. Moreover, by weakening the host defense, opportunistic periodontal pathogens, such as *P. gingivalis*, may allow the overgrowth of commensal oral species which leads to detrimental inflammation and periodontal bone loss [16].

## 2. The Junctional Epithelium-Bacteria Interactions

**Figure 1:**
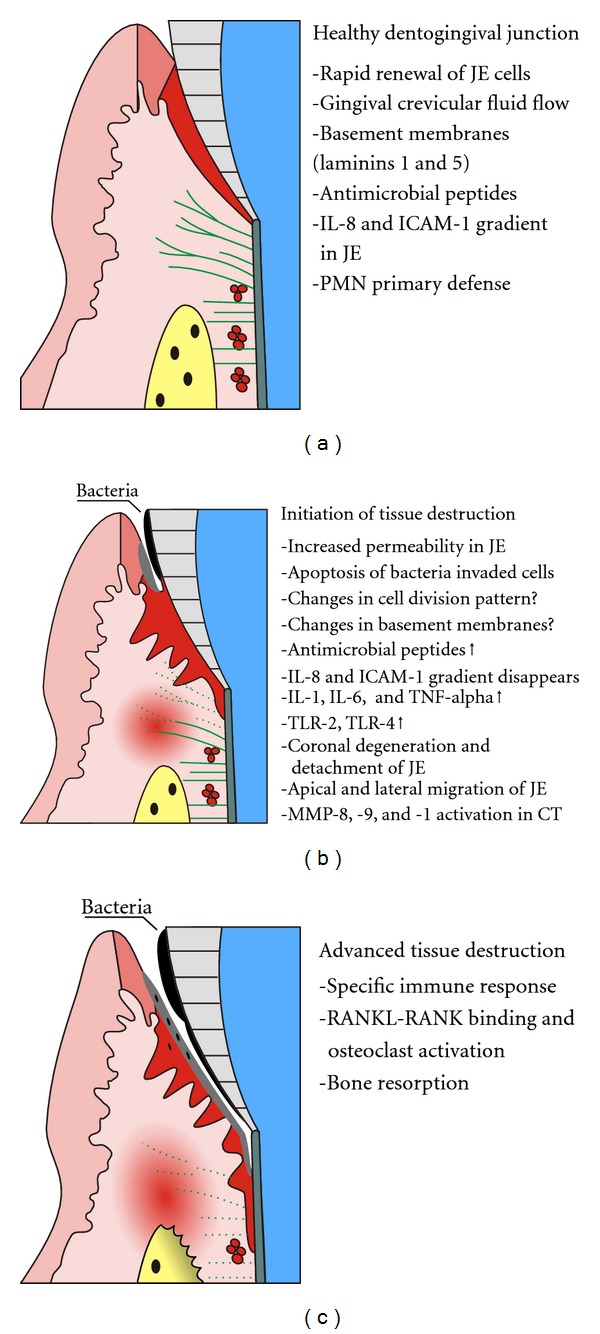
(a) Healthy dentogingival junction is an active part of the innate periodontal defense. (b) The epithelium and connective tissue are affected at the initial phase of periodontal tissue destruction. (c) In periodontitis the epithelial barrier is broken, bacteria may invade the tissue (black spots), and connective tissue and bone are degraded. Junctional epithelium, dark red. Connective tissue and periodontal ligament fibers green, and bone yellow. Bacteria black, pocket epithelium light gray. (a) Is a modified version of a figure from Pöllänen et al. [112].

The first line of innate host defense in the periodontal region is the JE that hinders bacterial advancement into periodontal tissues, Figure 1(a). JE is a unique epithelial structure firmly attached to the hard tissue of tooth *via* hemidesmosomes. The cells directly attached to the tooth, DAT-cells [17], have been shown to be dividing cells like the basal cells. Rapid renewal and constant shedding of the JE cells towards the sulcus together with the gingival crevicular fluid (GCF) flow are efficient inhibitors of bacterial colonization. This is further strengthened by the external basal lamina (EBL) and internal basal lamina (IBL) that function as barriers to bacterial advancement, yet allowing the passage of leukocytes and their antimicrobial agents and antibodies into the gingival crevice. Interestingly the composition of the IBL and EBL differs from each other and also seems to differ from the basement membrane of the outer gingival epithelium, suggesting a very different role for these basement membranes [18]. Whereas the IBL is dedicated to maintain the attachment to the tooth, the EBL functions merely as a protective barrier. The JE cells actively facilitate leukocyte recruitment to the site of inflammation by expressing chemotactic factors (IL-8 and complement C5a) and factors such as ICAM-1 that aid leukocyte course from the blood vessels [19–22]. The active role of the JE in the innate host defense is further demonstrated by the production of cytokines and the presence of natural antimicrobial peptides and proteins such as the defensins, the cathelicidin family members (LL-37), and calprotectin [23–25]. Human beta-defensins (hBDs) are expressed in gingival epithelia, salivary glands, saliva, and GCF [26–28] as a response to bacterial challenge [29–31]. Calprotectin, expressed in neutrophils, monocytes, and gingival keratinocytes, protects gingival keratinocytes against binding and invasion by *P. gingivalis* [32]. Alpha-defensins secreted by neutrophils are bound to junctional and pocket epithelium serving as an additional antimicrobial function [33]. It has been shown that JE cells lateral to DAT cells produce matrilysin (MMP-7) [34]. This enzyme is able to activate the precursor peptide of alpha-defensin, an important antimicrobial agent of mucosal epithelium [35]. It is possible that a similar active matrilysin/defensin system exists in JE, as in other mucosa exposed to bacteria such as intestine and lungs [36].

 A prerequisite for the bacteria to “win the battle” and gain more living space subgingivally appears to be degeneration of the DAT cells (Table 1). Alternatively, degradation of IBL on the tooth surface and consequently detachment of the JE takes place. A third possibility is bacterial invasion into JE cells and formation of an intraepithelial split that then results in periodontal pocket formation. These initial events in periodontal tissue destruction/pocket formation are surprisingly poorly known, Figure 1(b). Decreased mitosis and increased apoptosis of gingival epithelial cells has been shown at sites exhibiting severe inflammation [69]. Bacterial internalization in a tissue culture model and *in vivo* in severe periodontitis followed by epithelial cell apoptosis have also been demonstrated [70, 71]. A simultaneous application of LPS and proteases to rat gingiva has been found to cause apoptosis of connective tissue and periodontal ligament fibroblasts followed by apical migration of the JE [72].

During bacterial challenge the host cells recognize the bacteria and different antigens by different receptors that have been detected also on periodontal tissues. Toll-like receptor-2 (TLR-2), that recognizes, for example, bacterial peptidoglycans (PGN), lipoproteins, and LTAs has been found in abundance in the membrane of pocket epithelial cells as compared to the gingival tissues of healthy controls [73]. Protease activated receptors (PARs) mediate cellular responses to extracellular proteinases such as thrombin and trypsin-like serine proteases. PARs are expressed in human neutrophils, gingival fibroblasts, and osteoblasts [74, 75]. *In vitro* experiments have shown that *P. gingivalis* proteases stimulate PARs also in oral epithelial cells leading to proinflammatory cytokine IL-6 and IL-8 secretion [31, 76]. However, *P. gingivalis* may also have an opposite effect, since *P. gingivalis* invasion into epithelial cells has been shown to block IL-8 production and thus it may affect the chemotactic IL-8 gradient in the JE. [77, 78] *In vitro* studies have suggested that PAR-2 receptors expressed in gingival keratinocytes may contribute to hBD expression when challenged by gingipains, TNF-alpha or IL-1beta [31]. However, it seems that a certain threshold exists to inflammatory and bacterial challenge and the expression of hBDs, since in chronic (advanced) periodontitis the expression of hBDs-2 and -3 is reduced [28]. Application of PAR agonist peptide to gingiva has induced periodontitis in rats (radiographically assessed bone loss, myeloperoxidase (MPO) activity) [79]. Thus the activation of PARs seems to be important for the protective host response at the periodontal region. Whether modulation/activation/suppression of these cellular receptors can be used in periodontal therapy remains to be studied.

## 3. Connective Tissue (Fibroblasts and MMPs) Response to Bacterial Challenge

Gingival CT is composed of extracellular matrix (ECM) and fibroblasts producing the ECM and participating immune and inflammatory responses of the gingiva. The ECM comprises of fibers (mainly collagen I), proteoglycans, glycoproteins and water. CT is constantly renewed and that requires degradation of the ECM components, therefore gingival fibroblasts also produce proteolytic enzymes, MMPs. The MMPs are controlled by tissue inhibitors of MMPs (TIMPs) and in healthy periodontal tissue the TIMP levels exceed MMP [80]. Bacteria at the gingival margin causes drastic changes in CT; vascular permeability and the amount of inflammatory cell infiltrate are increased. Fibroblast functions are altered; cell proliferation and collagen production are impaired and components of extracellular matrix are degraded [81–84]. This is thought to result from both host- and bacteria-derived agents, such as lipopolysaccharides (LPSs), inflammatory cytokines, for example, IL-1beta, and TNF-alpha and to a lesser extent also by IL-17, growth factors and hormones [80, 85, 86] that activate leukocytes, fibroblasts, and epithelial cells leading to production of prostaglandins and MMPs and causing destruction of the CT [72, 87, 88]. In addition to the activation of the latent forms of MMPs, bacteria-derived proteinases may inactivate proteinase inhibitors such as alpha-1-antitrypsin and alpha-2-macroglobulin and thus result in higher levels of active proteinases [89–92]. Increased amounts of MMP-1, -2, -3, -8, -9, and -13 have been found in GCF and gingiva of periodontitis patients compared with samples from healthy controls [22, 85, 93–99]. Increased levels of MMPs and decreased amounts of their tissue inhibitors have been connected to the progression of periodontal disease [100, 101]. The destruction of CT in the periodontal region seems to be the result of synergistic action of both bacteria and host derived proteinases leading to an imbalance of the proteinases over their inhibitors [80]. Furthermore, a recent study on experimental periodontitis showed dual changes in CT; simultaneous collagen fiber breakdown and fiber bundle thickening, and suggested a protective role for the inflammatory tissue breakdown in order to avoid the spreading of the infection into the deeper areas [102].

## 4. Crosstalk between the Bacteria and  Polymorphonuclear-Leukocytes, Complement and Macrophages

PMNs are the main leukocytes isolated from gingival crevice. They are the first defense cells to respond to bacterial stimulus and they are present within minutes after stimulus. In the dentogingival area the bacteria are always present and the migration of PMNs and macrophages/monocytes into the gingival crevice is continuous. Thus even clinically healthy gingiva demonstrates some neutrophils in JE and the underlying CT [103]. Neutrophils are viable and functional in the gingival crevice. The “leukocyte wall” [104] is formed by neutrophils between the plaque and the junctional and sulcular epithelium. The leukocyte wall has proteolytic, phagocytic, and antibacterial (MPO, defensins, and other antibacterials such as lactoferrin) features. Although aimed against the bacteria, PMN activation and especially premature release of their granule products such as proteolytic enzymes and reactive oxygen species play a central role in periodontal tissue destruction [105, 106]. PMNs are essential in the first line of innate defense against plaque bacteria at the gingival margin. When the PMNs come to contact with the bacteria, they release their granular contents, adhere to individual bacteria and attempt to phagocytose them. The PMNs are a major contributor in the host parasite equilibrium but when activated can also cause tissue damage due to excess of enzymes, reactive oxygen species, MMPs, and other components that are released from their granules during the battle against microbes [48, 107–111].

Proteases such as elastase, cathepsin G, and MMPs, of which MMP-8, and MMP-9 are mainly originated from neutrophils, are able to degrade a wide variety of host cellular and extracellular components [112]. The fact, that when MMP activity is inhibited, tissue destruction is reduced, further emphasizes the role of MMPs on tissue destruction [113].

The main role of PMNs, however is phagocytosis and the PMNs in the gingival tissues are the main controllers of the microbial ecology within the gingival crevice. The cross-talk between neutrophils and immune cells is constant. Neutrophils have surface receptors for both complement (C5a-receptor) and immunoglobulin (Fc gamma-receptors) and the interaction with opsonized bacteria leads to phagocytosis of the bacteria. However, some periodontopathogens may escape PMN phagocytosis and even cause PMN death when ingested. Complement mediated phagocytosis of *A. actinomycetemcomitans *has been found inefficient regardless of the strain serotype, the majority of ingested *A. actinomycetemcomitans *staying viable. Furthermore, when opsonized with antibody, *A. actinomycetemcomitans* has caused rapid death of the PMNs [114]. Impaired PMN functions are generally considered to be related to periodontal tissue destruction and patients with reduced number of neutrophils or impaired neutrophil function show often severe periodontitis [115]. Smoking also impairs neutrophil functions, for example, phagocytosis [116]. However, some studies have indicated that, in contrast to impaired function, PMN hyperreactivity may play a role in periodontitis [117]. Increased oxygen radical and elastase release in response to bacterial stimulation has been connected, for example, to Fc gamma polymorphism (receptor RIIa131H/H genotype) [118]. The innate nature of PMN hyperresponsiveness is supported by a study showing high levels of PMN oxygen radical production in periodontitis patients also after periodontal treatment [27].

If the PMN dependent defense is insufficient, the inflammation is prolonged and lymphocytes, macrophages, and plasma cells start to dominate the infiltrate. This leads to the release of the proinflammatory cytokines (e.g., IL-1, IL-6, IL-8, and TNF-alpha) and prostaglandins (e.g., PGE_2_), and the adaptive immune response is set up. A shift in the balance between anti-inflammatory and proinflammatory cytokines may be crucial for the progression of periodontitis. TGF-beta is an anti-inflammatory cytokine that also inhibits epithelial cell proliferation. In healthy JE *α*v*β*6 integrin activates the latent TGF-beta. In inflamed pocket epithelium, however, *α*v*β*6 integrin is absent allowing high epithelial proliferation [119].

The nonspecific innate immune system provides an immediate response, recognizing the difference between self-components and foreign molecules in the periodontal region. The microbial recognition is based on interaction between microbial ligands known as pathogen-associated molecular patterns (PAMPs) (e.g., LPS, LTA, peptidoglycan (PGN), and fimbriae) and pattern-recognition receptors (PRRs) of host. Host PRRs may be either soluble (e.g., complement), membrane-bound (like TLRs, on the cells involved in immune response), or cytosolic (like nucleotide-binding oligomerization domain proteins, Nods, e.g., in epithelial cells lining mucosal surfaces and in phagocytes), for a review see: [37]. In most cases the interaction of PAMP with PRR leads to the production of proinflammatory cytokines and activation of inflammation reaction. However, various pathogenic bacteria including *P. gingivalis* may exploit the PRRs to undermine the bacterial killing, or use them to enable protected entry routs to host defense cells [120].

Complement system is the immediate and major humoral component of innate immune response [121]. It is activated immediately in the presence of a pathogen. The action of the complement system affects both innate and adaptive immunity and thus has an important role in inflammatory and immune responses. The complement system is a biochemical cascade of the small plasma proteins that activate another in series. Many of the complement proteins are proteases. They are stored and secreted as inactive proenzymes. Normally they are distributed throughout the body without adverse effects. At site of infection proenzymes become proteases and produce local inflammatory responses. There are three separate pathways of complement activation. The pathways include the classical pathway, the alternative pathway and the lectin-mediated pathway. The alternative pathway is activated directly by bacteria-C3 interaction, and the classical pathway by the antigen-antibody interaction with C4 [122–125]. The C3 fragment is important in all three pathways. In the periodontal region *T. denticola* has been shown to activate C3 by chymotrypsin like protease dentilisin and *P. gingivalis* both C3 and C5 by cysteine protease gingipain-1. This leads to formation of C3a, C3b, and C5a [126, 127]. The C3b molecule opsonizes pathogens and thus targets them to phagocytosis. C3a and also C5a in turn act as chemoattractants for phagocytes and activate mast cells. Mast cells contribute to the inflammatory reaction by releasing histamine, inflammatory mediators and cytokines such as leukotrienes. Mast cells show MMP-1, -2, and -8 activity which is linked to periodontal tissue breakdown [128]. Furthermore, complement activation increases vascular permeability and attracts phagocytes to the inflammatory site. In the periodontal region complement proteins and activation products have been detected in GCF and in the periodontal tissues both in health and disease [123, 129–131]. It has been suggested that complement deposits in connective tissue may reflect disease-associated complement activation [130]. Several activated complement components form a membrane attack complex (MAC), which creates a transmembrane pore leading to the lysis of the target cell and may thus lead to destruction of both bacterial and host cells. MAC inhibitor, CD59, is expressed in gingival epithelium and thus gingival epithelium is probably well-protected against MAC mediated cell damage [130]. Interestingly, the action of periodontal bacteria on complement seems to be biphasic. In addition to activation, periodontal pathogens such as *P. gingivalis *and *A. actinomycetemcomitans* may evade human complement mediated killing. Gingipains are proteases secreted by* P. gingivalis*. They degrade complement proteins. Other *P. gingivali*s proteinases inactivate leukocyte C5a receptor and *A. actinomycetemcomitans* serotype b lipopolysaccharide fails to interact with complement-derived opsonins [132–135]. In addition to the impaired pathogen-mediated complement activation, a clinical study has found partial gene deficiencies in complement C4 genes in patients suffering from chronic recurring periodontal inflammation [136]. C4 deficiencies have been previously associated with chronic mucosal infections and the authors suggested that it may also predispose to periodontal infections. However, the role of complement in periodontal diseases still needs further studies.

Precursors of the monocyte/macrophage lineage may differentiate into macrophages, dendritic cells, or osteoclasts [137]. Macrophages are efficient phagocytes and are abundant within the gingival tissues. They express TLRs that interact with PAMPs (LPS, LTA, and PGN) and release proinflammatory cytokines (IL-1, TNF-alpha, IL-6, and IL-12) and chemoattractants (e.g., IL-8). When differentiated into dendritic cells macrophages participate in antigen presentation by expressing costimulatory molecules and MHC-II molecules [37]. Dendritic cells form the crucial link between the innate and adaptive immunity. Langerin is a transmembrane cell surface protein which plays a role in antigen recognition and uptake. Langerhans cells, identified by the C-type lectin langerin, are dendritic cells responsible for the presentation of antigens to T-lymphocytes. In healthy gingival epithelium also large numbers of resident Langerin+ Langerhans cells have been found [137]. In periodontitis their number decreases intraepithelially and increases in connective tissue where antigen presentation takes place. The cells of the monocyte/macrophage lineage are important for the inflammatory reaction and also stand in the turning point of the adaptive host response. In addition, when differentiated to osteoclasts or via RANKL (receptor activator of nuclear factor kappa B-ligand) activation the monocyte-macrophage lineage cells also contribute to bone resorption [137]. Hyperreactive monocyte/macrophage IL-1 genotype has been connected to increased periodontal tissue destruction [138–140]. However, the connection between the IL-1 gene polymorphism and the clinical manifestations of periodontal disease do not seem to be likewise certain in all populations [141, 142].

## 5. The Role of the Specific (Adaptive) Immune Defense in Host-Bacteria Crosstalk

The innate immune system is a crucial part of the defense at the early stages of infection and further controls the emergence of the adaptive immune response [143] (Table 2). Periodontal pathogens may also evade or escape the innate defense mechanisms and in such cases the cells of the adaptive immune response have an important role in recognizing the pathogens and initiating specific defense targeted to the pathogens involved. The key cells of the adaptive immune defense are the T-lymphocytes (T-helper-1 (Th1), T-helper-2 (Th2), and T-killer-cells) and B-lymphocytes [144]. They have receptors (T-cell receptor, TCR, and B cell antigen receptor, BCR) on their surface that recognize and respond to bacterial antigens. Bacterial antigens coupled with antigen presenting cells with major histocompatibility complex (MHC) class-I molecules are recognized by T-killer cells and B cells. T-helper cells (Th1- and Th2-cells) recognize antigens coupled to class II MHC molecules. The recognition of the antigens in conjunction with costimulation with tumor necrosis factor (TNF) molecules leads to activation of the lymphocytes, production of the proinflammatory and/or anti-inflammatory cytokines (respectively Th1:IFN-gamma, IL-2, IL-12, and TNF-beta, Th2: IL-4, IL-5, IL-6 IL-10, and IL-13) and/or antibodies (by B cells; plasma cells/memory B cells). Both Immunoglobulin G and A (IgG and IgA) seem to be important to periodontal defense and are found in periodontal tissues and saliva [145–147]. During periodontal disease progression the levels of IgG and IgA against *A. actinomycetemcomitans* and *P. gingivalis* increase, and they correlate with the existing periodontitis and pathogen carriage in saliva [146, 147].

Although the immune response is primarily aimed to protect the host from the bacteria, it also seems to be strongly involved in tissue destruction in the periodontal region, Figure 1(c). Different models of tissue protection and destruction by the lymphocyte activation have been proposed [148–150] and the role of the different subsets of T-lymphocytes at different stages of the disease is still a matter of question. The anti-inflammatory cytokine profile of the Th2 response, low levels of costimulatory molecules and B-cell dependent antibody production have been suggested to dominate in cases of commensal bacteria, nonsusceptible patients and protective immune response whereas the periodontopathogenic bacteria are thought to trigger the proinflammatory Th1 cytokine response resulting in inflammatory periodontal bone resorption [88, 149–151]. Th1 cells express on their surface RANKL capable to bind to RANK on osteoclast precursor cells and thus directly induce osteoclastic bone resorption [152, 153]. Alternatively the cytokines produced by Th1 cells can indirectly lead to bone resorption by inducing RANKL expression on osteoblasts that can bind to RANK on osteoclast precursor cells and activate osteoclast differentiation [150]. However, different models have also been proposed where the Th2 cell response has been connected to periodontal bone destruction by uncontrolled B-cell production of IL-1 or B-cell expression of RANKL [152, 154]. Also osteoprotegerin (OPG), the known decoy receptor for and inhibitor of RANKL may regulate and protect the host from inflammatory bone resorption, since interference with RANKL by systemic administration of OPG has resulted in abrogation of periodontal bone resorption in a rat model [150]. Recent studies on humans have also suggested that in chronic periodontitis tissues a marked reduction in RANKL/OPG mRNA ratio may explain the immune response dependent bone resorption [155, 156].

## 6. *In Vitro* Models Studying Host-Microbe Interaction

Host-microbe interaction leading to periodontal pocket formation is a complex process, involving the presence of pathogenic bacterial biofilm as well as a susceptible host defense system. Improved methods to detect bacteria in periodontal biofilm have broadened our knowledge about pocket-associated microbes. In spite of the wide range of innate defense mechanisms at the dentogingival junction, the bacteria are able to surpass the host defense. Many of the periodontal pathogens take advantage of the host defense and thus enhance their growth in biofilm. It is clear that degradation of periodontal connective tissue and bone due to inflammation is an important part of the pocket formation. However, the most coronal part of the JE attached to the tooth appear to stand in first line of periodontal defense. Yet surprisingly small number of studies has been done on this early host-microbe interaction. The wide intercellular spaces and permeability of the JE, even though beneficial for the host defense, also allow bacteria and their harmful products to penetrate epithelium, which thereby activate the inflammatory reaction. The initial events of periodontal pocket formation are likely to involve destruction of the dento-epithelial junction resulting in the failure of the epithelial barrier function.

**Figure 2:**
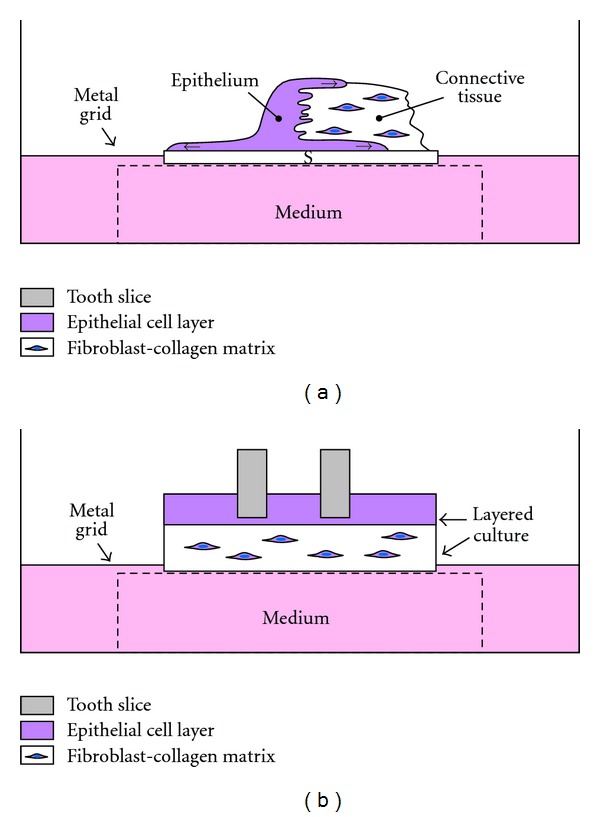
(a) A primary culture model for JE is formed when a piece of masticatory mucosa is placed on the top of a Millipore filter and cultured for 7–10 days. The interface between the filter and the epithelium shows morphologically similar hemidesmosomal attachment as the epithelium-tooth interface *in vivo*. Into this model planktonic bacteria or bacterial products can be added to the culture medium. (b) Organotypic culture model of the JE can be accomplished by culturing fibroblasts in a collagen gel and seeding keratinocytes and placing a piece of tooth on the top. Separately grown bacterial biofilms can be added onto the cultures. Cocultures with bacterial biofilms can be used to study host-microbe interactions with this model.

**Figure 3:**
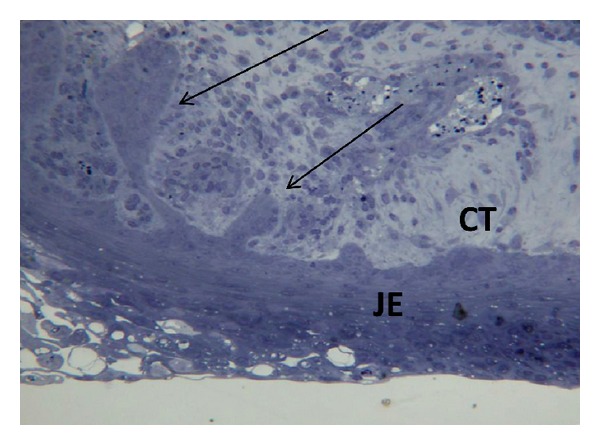
Primary JE culture treated with *A. actinomycetemcomitans *LPS shows migration of the epithelium into CT (arrows).

**Figure 4:**
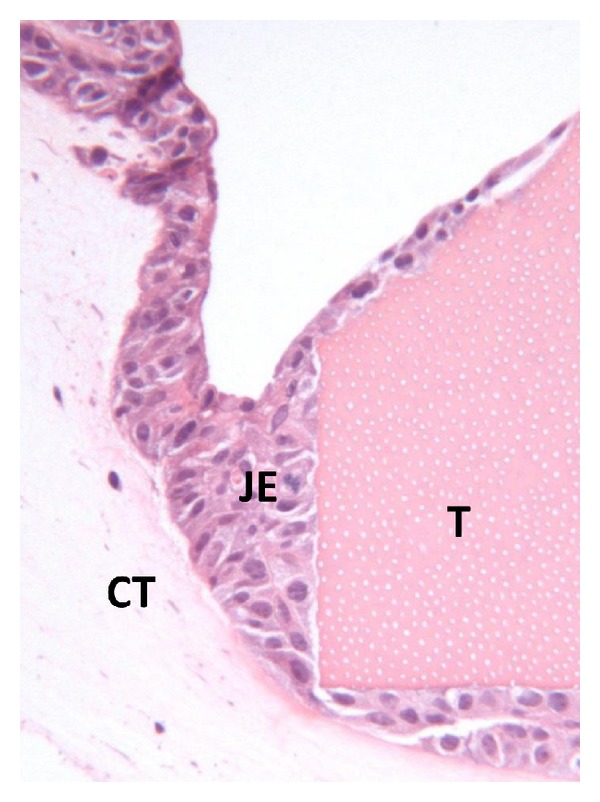
Organotypic culture model of the JE shows the structures of the dentogingival junction, JE = *in vitro* junctional epithelium, T = tooth, CT = connective tissue. When biofilm is placed on the top of the model host-bacteria interaction can be studied.

Monolayers of epithelial and connective tissue cells as well as planktonic bacteria have been widely used in studies on periodontal host-microbe interactions. Such experimental setups however lack important issues, for example, cell-cell contacts of the multilayered epithelium, the basement membranes and the epithelium-connective tissue interface as well as the microbial pathogenicity of the biofilm. As stated before in this paper, bacteria behave differently in biofilms, where some virulence genes are expressed due to the quorum sensing signaling. Different models of periodontal multilayer tissue cultures have been developed since the 1980s [157]. Two kinds of models have been used in our laboratory, Figures 2(a) and 2(b). The primary tissue culture model of JE Figure 2(a), in which a piece of masticatory mucosa is placed on a permeable filter, allows studies, for example, bacterial components on the dentogingival junction *in vitro*. Application of *A. actinomycetemcomitans* LPS into the culture medium has resulted in migration of the *in vitro* JE into the CT, Figure 3. Although this model is a very delicate and excellent model, it is difficult to repeat and individual variation may influence the results. The other model, Figure 2(b), originally presented by Oksanen and Hormia [158], uses cell lines of either oral or skin origin and further includes a piece of tooth onto which the JE is formed Figure 4. This model is easier to repeat and allows also the placement of bacterial biofilms on top of the culture. With this model *F. nucleatum* biofilm was shown to induce epithelial antimicrobial peptides human beta defensins 2 and 3 [159].

The organotypic tissue culture models are convenient systems to study how periodontal pathogens penetrate not only to the cells but also to the cell layers. *A. actinomycetemcomitans *passes through the gingival cell layers quickly intercellularly, whereas *P. gingivalis *invades the cell layer more slowly by using also intracellular route [160]. In addition, the biofilm mode of growth may alter the amount of cytokines, such as IL-1*β*, also called as “the gatekeeper of inflammation” for review see [161], in the host-bacterium contact site. Planktonic cells of opportunistic periodontal pathogens *A. actinomycetemcomitans* and *F. nucleatum* induce the IL-1*β* production from gingival epithelial cell multilayers [160]. However, at least *A. actinomycetemcomitans* biofilm has the capacity to bind and internalize IL-1*β* [162], which could slow down the progression of inflammation and modify the local microenvironment more favorable for bacterial growth.

## 7. Conclusions and Clinical Considerations

The host-microbe interaction at the dentogingival junction may involve several detrimental mechanisms leading to periodontal pocket formation.

### 7.1. Bacterial Enzymes Destroy the Dento-Epithelial Junction

It is probably not a coincidence that some of the known periodontopathogens, that is, *P. gingivalis* and *T. denticola* have strong proteolytic enzymes capable of degrading all the protein components as well as the proteins of cell surface and the cell-cell junctions. Morphological studies have shown that the IBL, can be detected on the tooth surface in advanced periodontitis adjacent to degenerated DAT cells [163]. However, this does not exclude the possibility that its molecular structures may be altered. Certain MMPs from eucaryotic cells are also able to cleave laminin-332, exposing a cryptic molecular site that triggers cell migration [164].

### 7.2. The Activated Epithelium Secretes Enzymes Degrading the Underlying Basement Membrane and Facilitates Epithelial Growth to Connective Tissue

Many bacterial products and cytokines are able to increase production of MMPs by epithelial cells. Since pathologically elevated levels of active MMPs have been found in periodontal tissues during periodontitis, the MMPs may play a role in lateral and apical migration of the JE during pocket formation [95, 165].

### 7.3. Adhesion of the Infected Epithelium to Tooth Is Weakened

 As an example, HSP60 from *A. actinomycetemcomitans* has been shown to decrease the number of epithelial basement membrane binding integrin, *α*6*β*4 [166]. This receptor is crucial for binding of JE to basement membrane. Absence of another integrin, *α*v*β*6, that activates TGF-beta, has been suggested to play a part in the formation of periodontitis [119].

### 7.4. PMNs Secrete Enzymes Degrading the Dentogingival Junction

 Many proteinases secreted by PMNs are capable of degrading basal lamina components, including laminin 332 [112]. In already early periodontitis a great number of PMNs are migrating through gingival epithelium.

### 7.5. Epithelial Cells of JE Undergo Degeneration and Apoptotic or Toxic Death

Periodontopathogens producing short chain fatty acids significantly impair the rapid renewal of the coronal JE/DAT cells and thereby counteract one of the tissues main host protective functions. Bacterial metabolites, LTAs, LPSs, and other toxins are able to cause epithelial cell death at high concentrations. Degeneration and detachment of the DAT cells, loss of cellular continuity, and cell death in the coronal part of the JE and development of an intraepithelial split have been suggested in the initial phase of periodontal pathology [167–170]. Studies have also reported apoptosis in cells of pocket epithelium caused by bacterial internalization [171] and enhanced activation of intracellular proteases that regulate apoptosis (caspases 3 and 7) [172].

The crucial steps in the failure of the host defense are the loss of the epithelial barrier function, the inflammation-driven degradation of the connective tissue and osteoclast activation via the imbalance of RANKL/OPG ratio. It has become evident, that both insufficient and exaggerated inflammatory response, are detrimental to host. The inhibited or exaggerated inflammatory response results from an imbalance of pro- and anti-inflammatory cytokines.

Although periodontal pocket formation/periodontitis is a pathologic process, we should keep in mind that, the host response that causes the tissue destruction, is aimed to restrict the spreading of the infection into deeper areas. Despite of a huge number of studies on periodontitis development, we still do not have a clear picture of the crucial events leading to the periodontal tissue destruction. It is obvious, however, that many factors, including direct bacterial effects and response of the host cells are involved in this process. Once these events are better understood treatment modalities aiming at preventing periodontal pocket formation can be designed. It should be emphasized, that when treating periodontal disease, continuous control of the infection should be the first goal, and only in addition to that, modulation of the host response may be considered.

## Figures and Tables

**Table 1:** Virulence factors of periodontopathogenic bacteria, and their effects on host cells.

Bacterial virulence factor	Effect on host cells	References
Fimbriae, flagellae	Promote bacterial colonization, adherence and invasion of host cells	[37, 38]
	Modulate inflammatory response	

Lipoteichoic acids (LTAs)	Mediate bacterial adhesion to human cells and teeth	[39–47]
	Arrest growth and decrease mitosis in epithelial cells	
	Stimulate leukocytes, activate complement	
	Increase cytokine and inflammatory mediator production	
	Stimulate bone resorption	

Lipopolysaccharides (LPSs)	Increase epithelial cell permeability, penetrate gingival epithelium	[39, 45, 48–58]
	Stimulate JE basal cell proliferation at high concentration (5000 *μ*g/mL)	
	Stimulate gingival fibroblast proliferation at low concentration (<10 *μ*g) and suppress at high concentration	
	Stimulate T-helper cell proliferation	
	Increase cytokine and inflammatory mediator production	
	Activate osteoclasts	

Short chain fatty acids (SCFAs)	Raise inflammatory response	[59–63]
	Inhibit gingival epithelial cell and fibroblast proliferation	

Proteinases	Activate host MMP:s, degrade extracellular matrix components, immunoglobulins and complement proteins	
	Promote apoptosis in gingival fibroblasts	[64]
	Induce human *β*-defensin-2 expression in gingival epithelial cells *in vitro *	

Heat shock proteins	Activate epithelial cells and osteoclasts at low concentrations and cause cell death at high concentrations	[65]

Cytolethal distending toxin	Upregulate RANKL expression in T cells	[66]

Leukotoxin	Cause apoptosis and necrosis of PMNs, T cells, natural killer cells	[48]

Capsule	Increase resistance to phagocytosis	[48]

Ammonium, hydrogen sulphide	Toxic to cells, cause cell vacuolization, inhibit collagen formation	[67, 68]

**Table 2:** Innate and adaptive defense in the periodontal region.

Innate defense response	Adaptive immune response
Epithelial barrier	Antigen presenting cells MHC-I, MHC-II
PMNs, complement	T helper 1 response
Monocyte/macrophage, mast cells	T helper 2 response
Fibroblasts	B cells, plasma cells
